# Impact of the number of cycles of platinum-based chemotherapy for early stage ovarian clear cell carcinoma on survival: a retrospective study

**DOI:** 10.1186/s12905-023-02405-0

**Published:** 2023-05-15

**Authors:** Yang Gao

**Affiliations:** 1 Department of Gynecological Oncology, Tianjin Central Hospital of Gynecology Obstetrics Affiliated to Nankai University, No. 156 Nankai San Ma Road, Tianjin, 300100 China; 2 Clinical College of Central of Gynecology and Obstetrics, Tianjin Medical University, Tianjin, China

**Keywords:** Chemotherapy cycles, Ovarian clear cell carcinoma, FIGO stage, Survival, Prognostic factor

## Abstract

**Background:**

Ovarian clear cell carcinoma (OCCC) is a unique subtype of ovarian epithelial ovarian cancer. The number of chemotherapy cycles for early-stage patients is still debated. This study aimed to evaluate whether at least 4 cycles of adjuvant platinum-based chemotherapy have better prognostic value than 1–3 cycles in early-stage OCCC.

**Methods:**

We retrospectively retrieved data from 102 patients with stage I-IIA OCCC between 2008 and 2017. All patients underwent complete surgical staging followed by adjuvant platinum-based chemotherapy. Kaplan-Meier curves and Multivariate Cox analysis were performed to estimate 5-year overall survival (OS) and progression-free (PFS) according to the number of chemotherapy cycles.

**Results:**

Among stage I-IIA disease, twenty (19.6%) patients received 1–3 cycles, and eighty-two (80.4%) patients received at least 4 cycles of adjuvant chemotherapy. Univariate analysis revealed that the patients in 1-3cycles group had not significantly improved 5-year OS and PFS than those in the ≥ 4 cycles group (5-year OS: hazard ratio [HR] 1.21; 95% confidence interval [CI] 0.25– 5.78, *p* = 0.1), and 5-year PFS: HR 0.79; 95% CI 0.26– 2.34, *p* = 0.1). In the multivariate analysis, there was no impact of 1–3 versus ≥ 4 cycles of chemotherapy on 5-year OS (HR 1.21, 95% CI 0.25–3.89, *p* = 0.8) or 5-year PFS (HR 0.94, 95% CI 0.32–2.71, *p* = 0.9). The potential independent risk factors associated with 5-year OS and PFS included the surgery approach and FIGO stage.

**Conclusion:**

The number of cycles of platinum-based chemotherapy could not be associated with a survival benefit for patients with early-stage OCCC.

## Background

Clear cell carcinoma of the ovary, a particular type of epithelial ovarian cancer (EOC), accounts for 5–30% of ovarian cancers [1]. Compared with other types of EOC, OCCC has a higher prevalence in Asia with a gradually increasing trend of young patients [2]. OCCC has unique clinical features and biological behavior. Due to the presentation of a large, unilateral, slow-growing pelvic mass, 60–70% of patients tend to be found on examination that the lesion is localized in the pelvic cavity (FIGO stage I-II) and has a better prognosis [3, 4]. However, the prognosis of patients with stage III-IV OCCC is worse than the serous ovarian subtype [5].

The National Comprehensive Cancer Network (NCCN) guidelines recommend that initial treatment of OCCC includes surgery and postoperative adjuvant chemotherapy regimens. Specifically, adjuvant chemotherapy should be offered to patients with all stages. Among them, observation can be considered for patients with stage IA without prospective data supporting the benefit of adjuvant chemotherapy [6]. Considering the high level of biological heterogeneity, including early-stage OCCC, the European Society of Medical Oncology (ESMO) guidelines recommend adjuvant chemotherapy for all patients regardless of sub-stage [7]. However, whether postoperative adjuvant chemotherapy is genuinely beneficial in patients with early-stage OCCC remains controversial, and there is no academic consensus on the optimal duration of adjuvant chemotherapy.

OCCC is not histologically a uniform type. Microscopically, clear, eosinophilic, and hobnail cells arranged in tubular cystic, papillary, and solid structures were typical morphological features of pure type [8]. Mixed types include heterogeneous mixtures such as endometrioid, mucoid, serous, and clear cell-like mixtures. For example, both endometrioid and clear cell carcinoma is closely related to endometriosis, but their pathogenesis is entirely different and belongs to different entities [9]. In addition, mixed OCCC with serous and clear cell components represents a similar stage, mitotic activities, and immunoreactivities to pure serous cancer [10]. Although mixed OCCC is uncommon, some scholars have proposed that pathologists should classify mixed histological types into separate, distinct biological and prognostic groups [11]. Previous studies [12, 13] might have been biased to a degree by including mixed histology. Based on the WHO classification of tumours of female reproductive organs (2020), excluding mixed types, a retrospective study focused on a homogenous population was designed to evaluate the impact of adjuvant chemotherapy cycles on survival in patients with early-stage OCCC.

## Materials and methods

### Study subjects

**Fig. 1:**
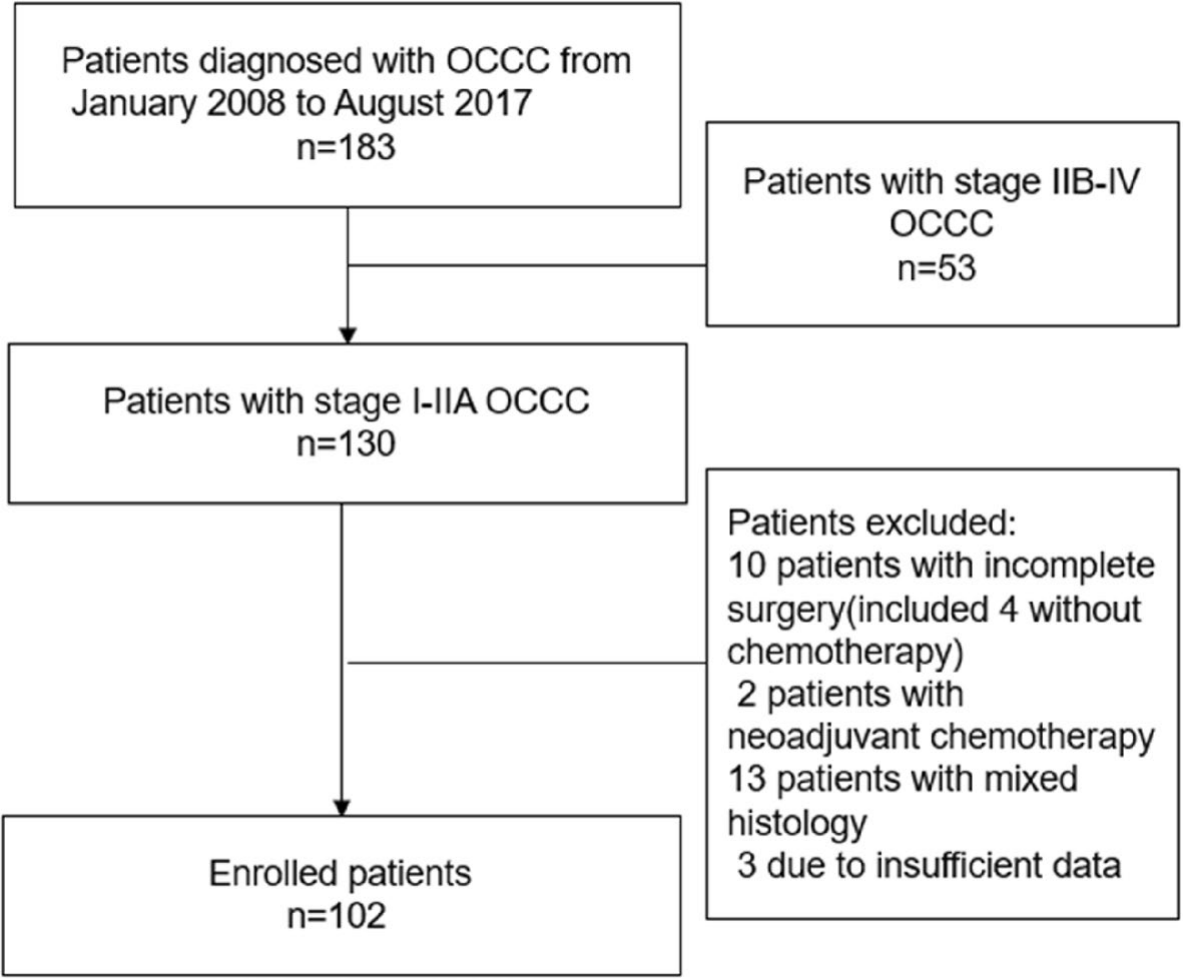
Flowchart of patients enrolled

We analyzed 102 early-stage (FIGOI-IIA) patients with pathologically confirmed OCCC at Tianjin Central Obstetrics and Gynecology Hospital between 2008 and 2017 (Fig. 1). Approval for this retrospective study was obtained from the Ethics Committee of the Tianjin Central Obstetrics and Gynecology Hospital (2017-KY98). Informed consent was obtained from all individual participants included in the study.

### Treatment and follow-up

All patients accepted initial standard complete surgical staging combined with postoperative chemotherapy with carboplatin (or cisplatin) plus paclitaxel. The treatment options called for paclitaxel (175 mg/m^2^) and carboplatin (area under the curve, 4 to 5) every 3 weeks. None of the patients underwent neoadjuvant chemotherapy or radiation before the initial operation. Regular follow-up included: gynecological examination, serum CA125 level, other tumor markers detection, and pelvic imaging assessment. The patients were followed until October 2022; the median follow-up duration was 61 months (9 ~ 165 months).

### Clinical data

One hundred two patients were divided into two groups based on postoperative treatment with chemotherapy cycles 1–3 or chemotherapy cycles ≥ 4. Clinicopathological data were extracted from the medical records, including age, menopause, family history of malignancy, tumor size, serum CA125, ascites positivity, endometriosis, surgical approach, and FIGO stage.

### Definition of relevant indicators

**Fig. 2:**
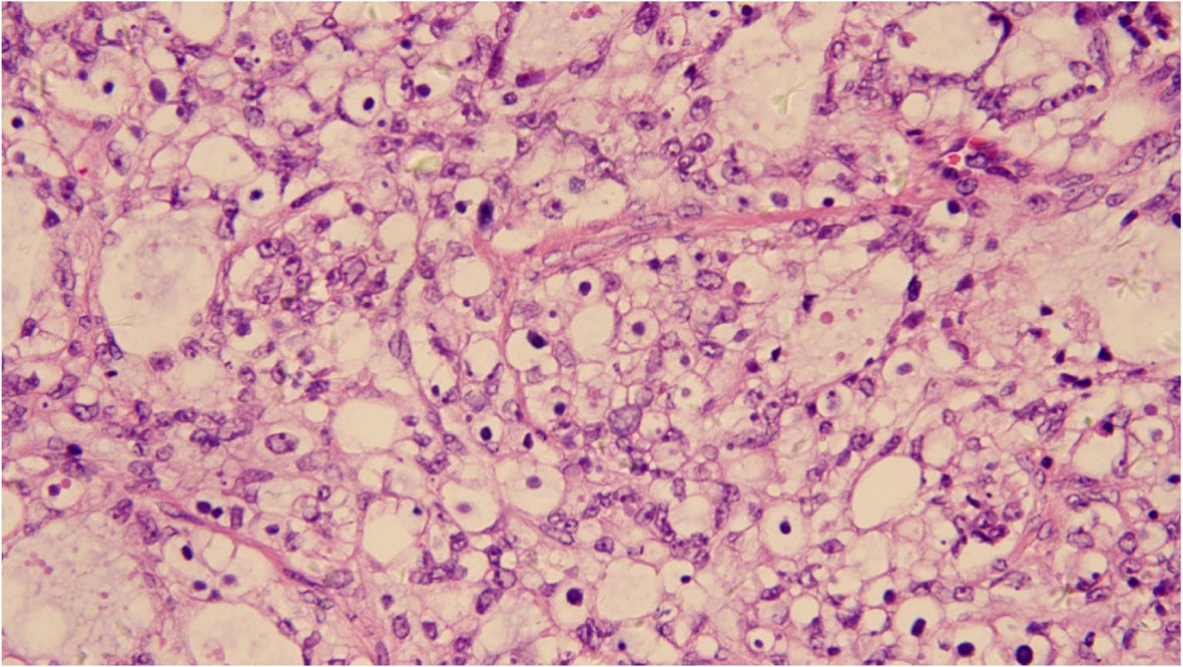
Clear cell carcinoma of ovary with clear and hobnail cells (H&E, original magnification x 40)

All patients were restaged according to FIGO 2014 staging system. Early-stage ovarian cancer was recognized as FIOG stage I-IIA [14]. Histologically, OCCC was characterized not only by tumor cell morphology but also by stromal features. Microscopically, clear and hobnail cells were the main features [15] (Fig. 2). Overall survival (OS) refers to the time from cancer diagnosis to death, and progression-free survival (PFS) refers to the time from cancer diagnosis to recurrence. Recurrence is characterized by the presence of the following two out of five conditions: (1) elevated serum CA125, (2) chest and abdominal effusion, (3) reoccurrence of abdominal and pelvic masses, (4) imaging findings, and (5) intestinal obstruction of unknown cause, after the condition is stable for more than six months postoperative chemotherapy.

### Statistical analysis

The χ2 test was used to compare the categorical variables, and the T-test was used to compare the continuous variables between the two groups. Kaplan-Meier curves were rendered to determine 5-year OS and PFS rates, while univariate analysis was performed using the log-rank test. Multivariate analyses for 5-year OS and PFS were completed using Cox regression models. All analyses were conducted using Statistical Analysis Software (SPSS version 27.0 for Windows (SPSS, Inc., Chicago, IL, USA). In all analyses, A *p*-value of < 0.05 was considered statistically significant.

## Result

One hundred two patients with FIGO stage I and IIA OCCC were identified. The overall clinical characteristics of patients are listed in Table 1. The average age at diagnosis was 51.71 ± 8.01 years. More than half of the patients were postmenopausal, had no family history of cancer, had tumors more significant than 10 cm in diameter, and were complicated with endometriosis. Ascites were cytologically negative in 85.3% of patients. 89.2% of patients underwent laparotomy complete surgical staging. According to FIGO standards, 46 patients were in stage IA, 0 in stage IB, 43 in stage IC, and 13 in stage IIA. Twenty patients received 1–3 cycles, and eighty-two received at least four cycles of chemotherapy. Among the 102 patients, there were 12 deaths and 19 recurrences. The overall 5-year OS and PFS rates were 88.2% and 81.4%, respectively.

**Table 1:** Clinicopathological characteristics

Parameters	All patients (*n* = 102) (n)	Percentage (%)
Age (yr)	51.71 ± 8.01	
≤ 52	49	48.0
> 52	53	52.0
Menopause		
Premenopause	35	34.3
Postmenopause	67	65.7
Family history of malignancy		
No	84	82.4
Yes	18	17.6
Tumor size		
<10cm	33	32.3
≥10cm	69	67.7
CA125 (u/ml)		
≤35	48	47.1
>35	54	52.9
Ascites positivity		
No	87	85.3
Yes	15	14.7
Endometriosis		
No	33	32.4
Yes	69	67.6
Surgical approach		
Laparotomy	91	89.2
Laparoscopy	11	10.8
FIGO		
IA	46	45.0
IB	0	0
IC	43	42.2
IIA	13	12.8
Chemotherapy cycle		
1-3	20	19.6
≥4	82	80.4

Clinico-pathological characteristics are summarized between the two groups in Table 2. It was more common for premenopausal women to receive 1–3 cycles of chemotherapy(55% versus 29.3%, *p* = 0.03). Between patients who received 1–3 cycles and ≥ 4 cycles of chemotherapy, there were no differences in age, family history of malignancy, tumor size, serum CA125, ascites positivity, endometriosis, surgical approach, or stage.

**Table 2:** Clinico-pathological characteristics of patients with early-stage OCCC who completed 1–3 or ≥ 4 cycles of platinum-based chemotherapy

Parameters	Chemotherapy cycle:1-3cycles (*n* = 20) (n,%)	Chemotherapy cycle: ≥4 cycles (*n* = 82) (n,%)	*P*
Age (yr)			
≤ 52	12 (60.0)	35 (42.7)	0.164
> 52	8 (40.0)	47 (57.3)	
Menopause			
Premenopause	11 (55.0)	24 (29.3)	0.030
Postmenopause	9 (45.0)	58 (70.7)	
Family history of malignancy			
No	15 (75.0)	69 (84.1)	0.525
Yes	5 (25.0)	12 (15.9)	
Tumor size			
<10cm	7 (35.0)	26 (31.7)	0.778
≥10cm	13 (65.0)	56 (68.3)	
CA125 (u/ml)			
≤ 35	7 (35.0)	41 (50.0)	0.228
> 35	13 (65.0)	41 (50.0)	
Ascites positivity			
No	15 (75.0)	72 (87.8)	0.272
Yes	5 (25.0)	10 (12.2)	
Endometriosis			
No	7 (35.0)	26 (31.7)	0.778
Yes	13 (65.0)	56 (68.3)	
Surgical approach			
Laparotomy	15 (75.0)	76 (92.7)	0.060
Laparoscopy	5 (25.0)	6 (7.3)	
FIGO			0.182
IA	9 (45.0)	37 (45.1)	
IC	6 (30.0)	37 (45.1)	
IIA	5 (25.0)	9 (9.8)	

**Fig. 3:**
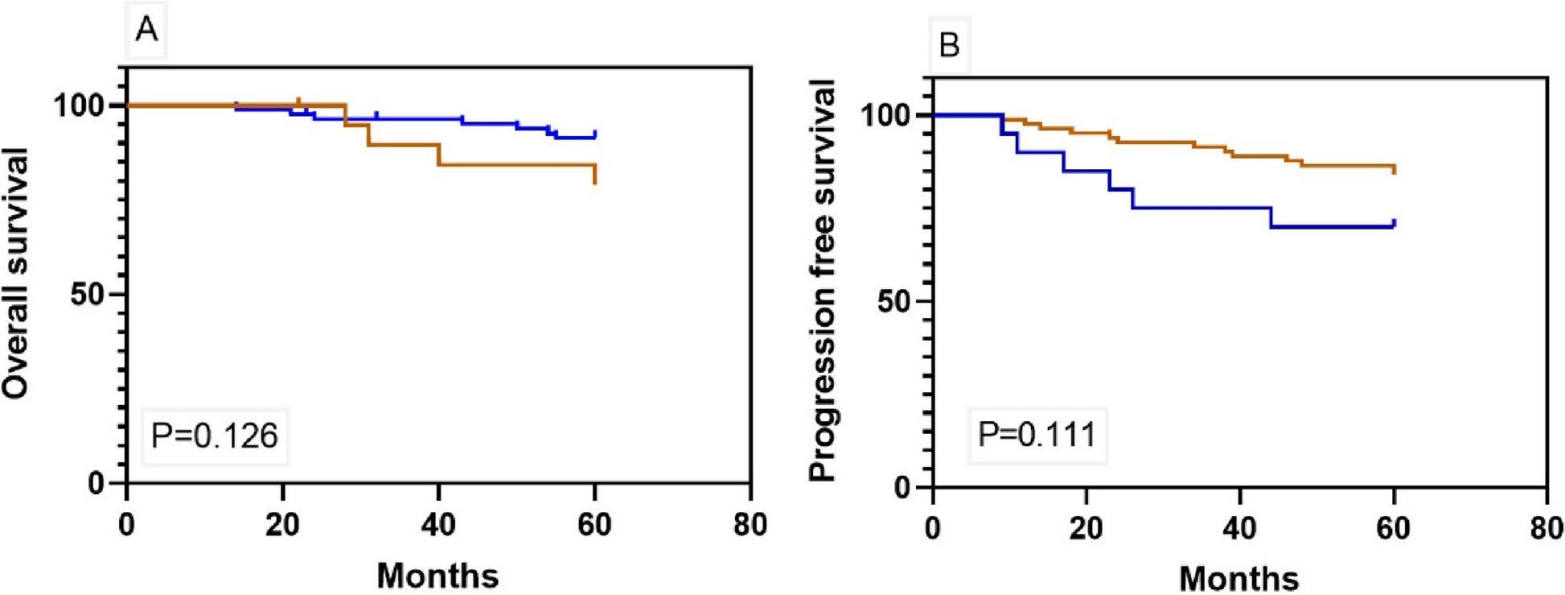
Kaplan-Meier curves of (**A**) 5-year OS and (**B**) 5-year PFS among patients with stage I-IIA OCCC who completed 1–3 or at least 4 cycles of platinum-based chemotherapy (blue, 1–3 cycles, *n* = 20; brown, ≥ 4 cycles, *n* = 82, respectively)

**Fig. 4:**
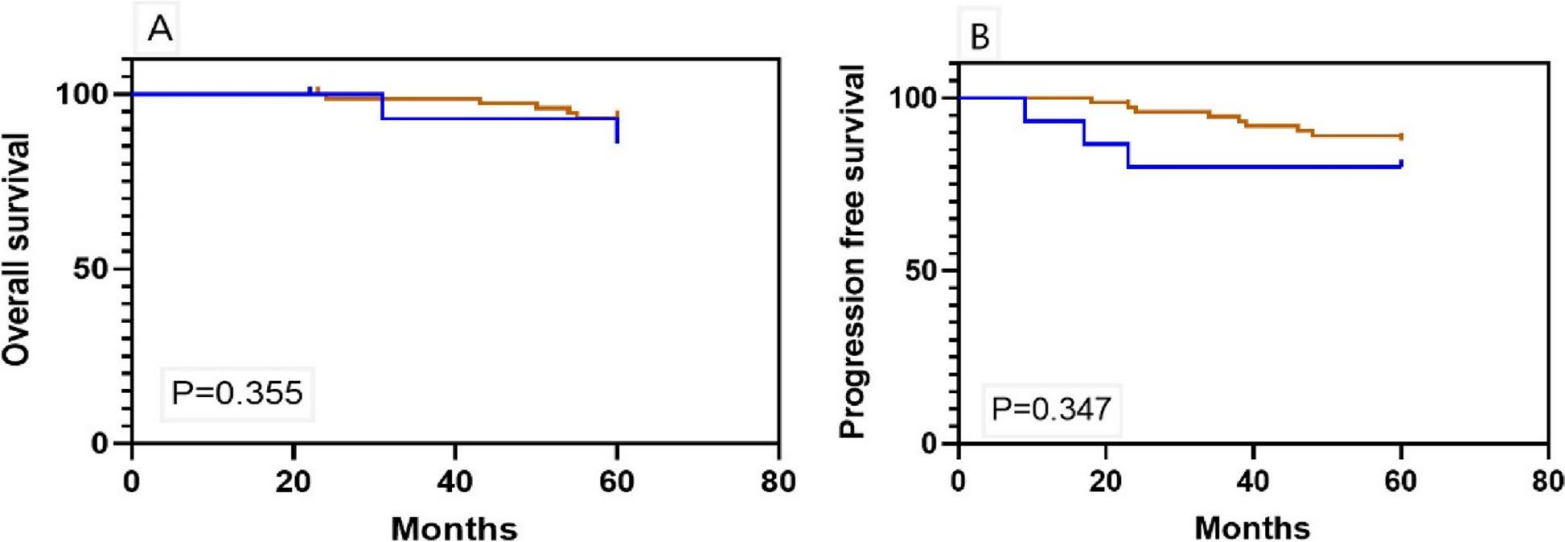
Kaplan-Meier curves of (**A**) 5-year OS and (**B**) 5-year PFS among patients with stage I OCCC who completed 1–3 or at least 4 cycles of platinum-based chemotherapy (blue, 1–3 cycles, *n* = 20; brown, ≥ 4 cycles, *n* = 82, respectively)

Figure 3 shows the Kaplan-Meier curves for 5-year OS and PFS among early-stage patients. The duration of adjuvant chemotherapy was not associated with improved 5-year OS (HR 1.21; 95% CI 0.25–5.78, *p* = 0.1) or PFS (HR 0.79; 95% CI 0.26–2.34, *p* = 0.1). A subgroup analysis of substages was performed. Figure 4 demonstrates the Kaplan-Meier curves for 5-year OS and PFS among stage I. Those who received 1–3 cycles or at least four cycles of chemotherapy had similar survival outcomes (5-year OS, HR 1.39; 95% CI 0.76–3.34, *p* = 0.4; 5-year PFS, HR1.58; 95% CI 0.46–4.34, *p* = 0.3).

The univariate analysis for 5-year OS revealed endometriosis (HR0.17, 95%CI 0.36–0.78, *p* = 0.02), surgical approach (HR9.69, 95%CI 1.14–10.25, *p* = 0.04) and FIGO stage (HR4.91, 95%CI 1.53–8.43, *p* = 0.02) were significant factors. There were insignificant differences in age, menopause status, family history of malignancy, tumor size, serum CA125, and ascites cytology. After adjustment by multivariate analysis, chemotherapy cycles were still non-significant (HR 1.21, 95% CI 0.25–3.89, *p* = 0.8). Laparoscopic approach (HR 8.54, 95% CI 1.73–11.78, *p* = 0.01) and advancing stage (HR 6.94, 95% CI 1.25–9.78, *p* = 0.01) were associated with poorer 5-year OS (Table 3).

**Table 3:** Cox proportional hazards model of 5-year OS with early-stage OCCC

Parameters	HR	95%CI	*P*-value
Endometriosis (with vs. without)	0.39	0.15–1.12	0.055
Surgical approach (laparotomy vs. laparoscopy)	8.54	1.73–11.78	0.009
FIGO stage (IA vs. IC vs. IIA)	6.94	1.25–9.78	0.012
Chemotherapy cycle (1–3 vs. ≥4)	1.21	0.25–3.89	0.778

IA is a referential parameter

In the univariate analysis for 5-year PFS, there were statistically significant in endometriosis (HR0.37, 95%CI 0.13–0.98, *p* = 0.046), surgical approach (HR4.15, 95%CI 1.07–12.08, *p* = 0.04) and FIGO stage (HR7.42, 95%CI 1.90–10.99, *p* = 0.004). No differences were observed in age, menopause status, family history of malignancy, tumor size, serum CA125, and ascites cytology. The multivariate analysis showed that chemotherapy cycles remained non-significant (HR 0.94, 95% CI 0.32–2.71, *p* = 0.9). Surgical approach (HR4.13, 95%CI 1.18–10.48, *p* = 0.03) and FIGO stage (HR6.40, 95%CI 2.1–9.15, *p* < 0.001) were independently associated with 5-year PFS (Table 4).

**Table 4:** Cox proportional hazards model of 5-year PFS with early-stage OCCC

Parameters	HR	95%CI	*P*-value
Endometriosis (with vs. without)	0.44	0.18–1.32	0.080
Surgical approach (laparotomy vs. laparoscopy)	4.13	1.18–10.48	0.027
FIGO stage (IA vs. IC vs. IIA)	6.40	2.1–9.15	< 0.001
Chemotherapy cycle (1–3 vs. ≥4)	0.94	0.32–2.71	0.903

## Discussion

In this work, we rule out the mixed histology to avoid possible bias and find that neither the 1–3 cycles nor at least four cycles of chemotherapy influence the 5-year OS or PFS of early-stage OCCC patients.

The prognostic implications of the number of adjuvant chemotherapy in patients with OCCC remain controversial. 3–6 cycles of chemotherapy are usually administered after the initial surgery for ovarian cancer. Prolonged chemotherapy cycles may contribute to eradicating residual lesions and improve the insufficiency of three courses [16]. Whereas another multicenter retrospective study to early-stage OCCC revealed that patients who received 3 or 6 cycles of chemotherapy experienced similar survival [13].

Recommendations of greater efficacy for more cycles of postoperative adjuvant chemotherapy were derived from the results obtained in advanced-stage ovarian cancer [17]. Few studies have been reported concerning the optimal number of chemotherapy cycles on early-stage cancer and histological subtype. In the Gynecologic Oncology Group (GOG) 157 trial, 427 patients with high-risk early-stage ovarian cancer completed adjuvant carboplatin and paclitaxel regimens. The results showed that six cycles of chemotherapy reduced the recurrence rate in patients with serous ovarian cancer compared with three cycles. However, it did not apply to OCCC [12]. The OCCC patients can not benefit from multicycle chemotherapy, perhaps because early-stage OCCC has a better prognosis than the serous subtype [18]. Only 30% of OCCC patients were enrolled in the GOG157 trial. Another multicenter retrospective study included 210 patients with stage I-II OCCC with similar conclusions. There was no significant difference in PFS and OS between the chemotherapy group’s three and six cycles [13]. Similarly, Wang et al. reported that the prognosis of patients who underwent > 4 cycles of chemotherapy might not be improved in stage I OCCC [19].

This study did not demonstrate that OCCC patients could benefit from the different number of adjuvant chemotherapy cycles in either the early-stage cohort or stage I cohort. Although most Kaplan-Meier curves showed a trend of better prognosis after at least four courses, there was no statistical difference. The same conclusion was obtained by removing confounding factors through a multivariate analysis. The reason may be the characteristics of platinum-based chemotherapy insensitivity due to the slow growth and long cell doubling time of OCCC in the early stage [20]. At some point, studies of different Combined chemotherapy protocols or target therapies were urgently needed to ensure benefit from chemotherapy in patients of early-stage OCCC.

It was well-known that the FIGO stage influenced the prognosis of OCCC. This study confirmed this accepted theory. In addition, our findings showed that the surgical approach was an independent prognostic factor of early-stage OCCC. Laparotomy had a better 5-year PFS and OS compared with laparoscopy. OCCC was often complicated with endometriosis cyst and severe adhesion, leading to iatrogenic tumor rupture and upgraded stage in laparoscopic surgery. Some scholars mentioned that laparoscopic surgery was comparable to open surgery in the accuracy and thoroughness of complete surgical staging for early ovarian cancer. However, the oncologic safety needed to be further demonstrated [21]. However, the retrospective data from Yin et al. proposed that laparoscopic surgery did not affect the PFS and OS of early-stage OCCC [22]. So a well-designed randomized controlled trial was warranted.

The limitations of this present study are retrospective nature and single-center analysis, potentially raising the chance of sectional bias. Another caveat is the limited number of participants and end-point events, affecting the estimation accuracy of the prognosis. However, unlike the previous research, homogenous histology is adopted. In addition, the entire cohort is treated with standard complete surgical staging followed by platinum-based chemotherapy combined with paclitaxel.

## Conclusion

In conclusion, the recurrence and survival outcomes for 1–3 chemotherapy cycles were similar to that for at least four cycles as an adjuvant protocol for early-stage OCCC. Given the adverse effects of chemotherapy on patients’ quality of life, the decision of chemotherapy cycles should be individualized after balancing the risk and benefits. Specifically, laparotomy may be an optimal surgery approach to improve prognosis. Further prospective validations are needed to elucidate the best treatment option for early-stage OCCC.

## Data Availability

The datasets used and analyzed during the current study are available from the corresponding author upon reasonable request.

## Abbreviations

CA-125: Cancer antigen 125
CI: Confidence interval
EOC: Epithelial ovarian carcinoma
ESMO: European Society of Medical Oncology
FIGO: International Federation of Gynecology and Obstetrics
HR: Hazard ratio
NCCN: National Comprehensive Cancer Network
OCCC: Ovarian clear cell carcinoma
OS: Overall survival
PFS: Progression-free survival

## References

[1] Zhu C, Xu Z, Zhang T, Qian L, Xiao W, Wei H, Jin T, Zhou Y (2021). Updates of Pathogenesis, Diagnostic and therapeutic perspectives for ovarian Clear Cell Carcinoma. J Cancer.

[2] Machida H, Matsuo K, Yamagami W, Ebina Y, Kobayashi Y, Tabata T, Kanauchi M, Nagase S, Enomoto T, Mikami M (2019). Trends and characteristics of epithelial ovarian cancer in Japan between 2002 and 2015: a JSGO-JSOG joint study. Gynecol Oncol.

[3] Winterhoff B, Hamidi H, Wang C, Kalli KR, Fridley BL, Dering J, Chen HW, Cliby WA, Wang HJ, Dowdy S (2016). Molecular classification of high grade endometrioid and clear cell ovarian cancer using TCGA gene expression signatures. Gynecol Oncol.

[4] Yamashita Y (2015). Ovarian cancer: new developments in clear cell carcinoma and hopes for targeted therapy. Jpn J Clin Oncol.

[5] Liu H, Xu Y, Ji J, Dong R, Qiu H, Dai X (2020). Prognosis of ovarian clear cell cancer compared with other epithelial cancer types: a population-based analysis. Oncol Lett.

[6] Armstrong DK, Alvarez RD, Bakkum-Gamez JN, Barroilhet L, Behbakht K, Berchuck A, Chen LM, Cristea M, DeRosa M, Eisenhauer EL (2021). Ovarian Cancer, Version 2.2020, NCCN Clinical Practice Guidelines in Oncology. J Natl Compr Canc Netw.

[7] Colombo N, Sessa C, du Bois A, Ledermann J, McCluggage WG, McNeish I, Morice P, Pignata S, Ray-Coquard I, Vergote I (2019). ESMO-ESGO consensus conference recommendations on ovarian cancer: pathology and molecular biology, early and advanced stages, borderline tumours and recurrent diseasedagger. Ann Oncol..

[8] Bennett JA, Dong F, Young RH, Oliva E (2015). Clear cell carcinoma of the ovary: evaluation of prognostic parameters based on a clinicopathological analysis of 100 cases. Histopathology.

[9] Fadare O, Parkash V (2019). Pathology of Endometrioid and clear cell carcinoma of the Ovary. Surg Pathol Clin.

[10] Han G, Gilks CB, Leung S, Ewanowich CA, Irving JA, Longacre TA, Soslow RA (2008). Mixed ovarian epithelial carcinomas with clear cell and serous components are variants of high-grade serous carcinoma: an interobserver correlative and immunohistochemical study of 32 cases. Am J Surg Pathol.

[11] Cuff J, Longacre TA (2012). Endometriosis does not confer improved prognosis in ovarian carcinoma of uniform cell type. Am J Surg Pathol.

[12] Chan JK, Tian C, Fleming GF, Monk BJ, Herzog TJ, Kapp DS, Bell J (2010). The potential benefit of 6 vs. 3 cycles of chemotherapy in subsets of women with early-stage high-risk epithelial ovarian cancer: an exploratory analysis of a gynecologic Oncology Group study. Gynecol Oncol.

[13] Prendergast EN, Holzapfel M, Mueller JJ, Leitao MM, Gunderson CC, Moore KN, Erickson BK, Leath CA, Diaz Moore ES, Cohen JG (2017). Three versus six cycles of adjuvant platinum-based chemotherapy in early stage clear cell ovarian carcinoma – a multi-institutional cohort. Gynecol Oncol.

[14] Lorusso D, Pignata S (2017). Role of adjuvant chemotherapy in early-stage endometrioid and clear-cell ovarian cancer. Ann Oncol.

[15] Kato N (2020). Pathology of clear cell carcinoma of the ovary: a basic view based on cultured cells and modern view from comprehensive approaches. Pathol Int.

[16] Chung YS, Kim YJ, Lee I, Lee JY, Nam EJ, Kim S, Kim SW, Kim YT (2017). Impact of neoadjuvant chemotherapy and postoperative adjuvant chemotherapy cycles on survival of patients with advanced-stage ovarian cancer. PLoS ONE.

[17] Lee SJ, Lee JW, Min JA, Park CS, Kim BG, Lee JH, Bae DS (2006). A pilot study of three-cycle consolidation chemotherapy with paclitaxel and platinum in epithelial ovarian cancer patients with clinical complete response after paclitaxel and platinum chemotherapy. Int J Gynecol Cancer.

[18] Mizuno M, Kajiyama H, Shibata K, Mizuno K, Yamamuro O, Kawai M, Nakanishi T, Nagasaka T, Kikkawa F (2012). Adjuvant chemotherapy for stage i ovarian clear cell carcinoma: is it necessary for stage IA?. Int J Gynecol Cancer.

[19] Wang T, Zeng J, Li N, Zhang R, Song Y, Wu L (2020). An exploratory analysis about cycles of adjuvant chemotherapy and outcomes by substage for stage I ovarian clear cell carcinoma: a single institution retrospective study. Am J Cancer Res..

[20] Itamochi H, Kigawa J, Akeshima R, Sato S, Kamazawa S, Takahashi M, Kanamori Y, Suzuki M, Ohwada M, Terakawa N (2002). Mechanisms of cisplatin resistance in clear cell carcinoma of the ovary. Oncology.

[21] Park JY, Bae J, Lim MC, Lim SY, Seo SS, Kang S, Park SY (2008). Laparoscopic and laparotomic staging in stage I epithelial ovarian cancer: a comparison of feasibility and safety. Int J Gynecol Cancer.

[22] Yin S, Gao W, Shi P, Xi M, Tang W, Zhang J (2021). Primary laparoscopic surgery does not affect the prognosis of early-stage ovarian clear Cell Cancer. Cancer Manag Res.

